# Leukocyte-Mediated Cardiac Repair after Myocardial Infarction in Non-Regenerative vs. Regenerative Systems

**DOI:** 10.3390/jcdd9020063

**Published:** 2022-02-21

**Authors:** Elizabeth Anne Peterson, Jisheng Sun, Jinhu Wang

**Affiliations:** Division of Cardiology, School of Medicine, Emory University, Atlanta, GA 30322, USA; elizabeth.anne.peterson@emory.edu (E.A.P.); jisheng.sun@emory.edu (J.S.)

**Keywords:** leukocyte, immune cells, humans, mice, zebrafish, heart, cardiac injury, myocardial infarction, inflammatory, repair, regeneration

## Abstract

Innate and adaptive leukocytes rapidly mobilize to ischemic tissues after myocardial infarction in response to damage signals released from necrotic cells. Leukocytes play important roles in cardiac repair and regeneration such as inflammation initiation and resolution; the removal of dead cells and debris; the deposition of the extracellular matrix and granulation tissue; supporting angiogenesis and cardiomyocyte proliferation; and fibrotic scar generation and resolution. By organizing and comparing the present knowledge of leukocyte recruitment and function after cardiac injury in non-regenerative to regenerative systems, we propose that the leukocyte response to cardiac injury differs in non-regenerative adult mammals such as humans and mice in comparison to cardiac regenerative models such as neonatal mice and adult zebrafish. Specifically, extensive neutrophil, macrophage, and T-cell persistence contributes to a lengthy inflammatory period in non-regenerative systems for adverse cardiac remodeling and heart failure development, whereas their quick removal supports inflammation resolution in regenerative systems for new contractile tissue formation and coronary revascularization. Surprisingly, other leukocytes have not been examined in regenerative model systems. With this review, we aim to encourage the development of improved immune cell markers and tools in cardiac regenerative models for the identification of new immune targets in non-regenerative systems to develop new therapies.

## 1. Introduction

**Figure 1:**
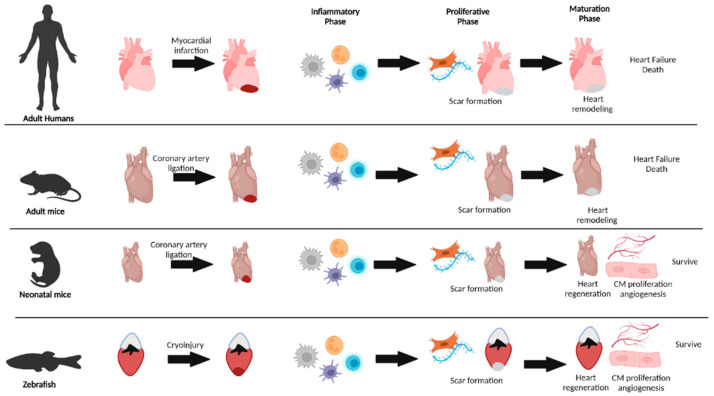
**Comparison of cardiac tissue repair in non-regenerative vs. regenerative systems.** Schematic demonstrating the three phases (inflammatory, proliferative, and maturation) of heart tissue repair in humans after myocardial infarction or in animal models after cardiac injury. All systems undergo rapid inflammatory phases, but differences occur in the duration of this phase in non-regenerative vs. regenerative model systems. Extensive inflammation (weeks to months) observed in adult mice and humans contributes to permanent scar formation and heart remodeling, eventually leading to heart failure and death. Complete cardiac regeneration occurs in neonatal mice (21 days) and zebrafish (60 days) models with timely resolution of the inflammatory phase (within 1 week) before scar formation in the proliferative phase and then scar resolution in the maturation phase with cardiomyocyte proliferation and angiogenesis. Created with BioRender.com (accessed on 8 February 2022).

Cardiovascular disease (CVD) persists as the primary disease burden in the United States, with nearly 659,000 people dying each year and costing 363 billion USD [1,2]. During myocardial infarction (MI), ischemic myocardial fibers become necrotic and release damage-associated molecular patterns (DAMPs) and alarmins [3]. These molecules rapidly mobilize an arsenal of inflammatory leukocytes such as neutrophils, monocytes/macrophages, and dendritic cells to the ischemic area for the removal of dead cells and cellular debris, and disintegration of the extracellular matrix (ECM). Macrophages then clear apoptotic neutrophils to induce inflammation resolution and support the transition to a proliferative phase [4]. During this next phase, myofibroblasts generate granulation tissue, and endothelial cells differentiate to form new blood vessels [5]. In non-regenerative adult mammals, the maturation phase of tissue repair forms a non-contractile scar at the injury site, contributing to heart failure development and patient death [6,7,8] (Figure 1). As recent studies demonstrated low rates of cardiomyocyte renewal throughout life in humans and cardiac regeneration capabilities in mammalian neonates [9,10,11,12,13,14], researchers are actively searching for factors capable of stimulating endogenous regeneration mechanisms in humans by examining innate regenerative responses in other vertebrate animal models, such as zebrafish and neonatal mice [15,16,17].

The zebrafish is a well-established model organism for tissue regeneration, due to its fully sequenced genome, large percentage of orthologs to human genes, and similar anatomy and cell types to humans [18,19]. Zebrafish fully regenerate their lost tissues without scar formation after cardiac injury by ventricle resection, cryoinjury, and cardiomyocyte genetic ablation [20,21,22,23,24]. During zebrafish heart regeneration, pre-existing cells serve as the source for a new myocardium, epicardium, and endocardium [25,26,27,28]. Importantly, zebrafish studies identified essential molecules for regeneration, some of which induced cardiac regeneration in mammals [28,29,30,31,32,33,34,35,36,37,38,39]. Within the first two days of life, neonatal mammals also demonstrate vigorous regenerative responses after cardiac injury with ventricular resection or ligation of the left anterior descending (LAD) coronary artery [40,41,42]. As with adult zebrafish, neonatal mammalian cardiac regeneration involves expansion of pre-existing cardiomyocytes, endothelial cells, and epicardial cells for fibrotic scar clearance (Figure 1) [40,43,44,45]. Although inflammation after cardiac injury in adult mammals contributes to cytotoxicity and fibrotic scar formation [46], inflammation is required for cardiac regeneration in both adult zebrafish and neonatal mice [47,48] (Table 1 and Table 2). Moreover, leukocytes demonstrated essential roles for regenerative events such as CM proliferation, revascularization, collagen deposition, and scar resolution (Table 1). For further information on zebrafish and neonatal heart regeneration, please see the following reviews [13,14,18,49].

**Figure 2:**
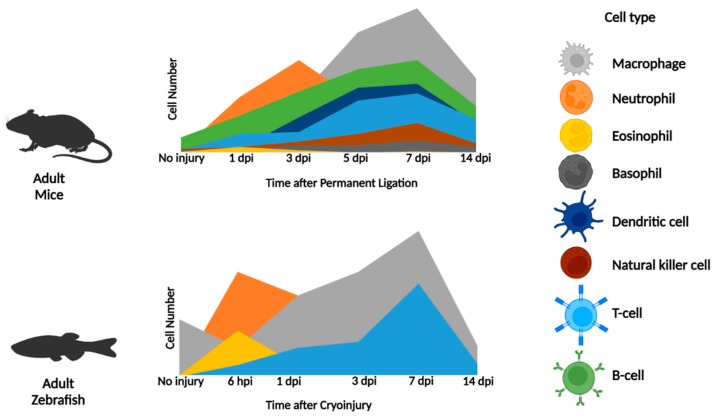
**Leukocyte recruitment after cardiac injury in non-regenerative vs. regenerative animal model systems.** Schematic of immune cell mobilization in myocardial infarction injury models after left anterior descending (LAD) coronary artery ligation in non-regenerative adult mice (upper) and cryoinjury in regenerative adult zebrafish (lower). Adult mice schematic adapted from previously published data [58,125,126]. Adult zebrafish schematic adapted from previously published data [68]. No injury represents steady-state levels of leukocytes without heart injury; hpi stands for hours post-injury; dpi stands for day(s) post-injury. At present, basophils, dendritic cells, natural killer cells, and B cells have no specific marker or antibody to label these cells in the zebrafish model which explains the lack of these cell types in the representative schematic. Created with BioRender.com (accessed on 8 February 2022).

As regenerative systems contain the same immune cell types as non-regenerative organisms and require timely inflammation and resolution for effective regeneration [18,19], we can characterize the immune response during regeneration to discover new therapeutic targets. This review relates what is currently known about immune cells, particularly neutrophils, macrophages, and T cells, after heart damage in non-regenerative systems such as humans and adult rodents with regenerative neonatal mice and adult zebrafish (Figure 2, Table 1). Information on the role of other leukocytes such as eosinophils, basophils, dendritic cells, natural killer cells, and B cells after cardiac injury in non-regenerative systems is also provided (Table 2). However, at present, these cell types have not been examined in a cardiac regenerative environment, and whether differences occur in regenerative vs. non-regenerative systems remains unknown.

## 2. Leukocytes Examined in Non-Regenerative and Regenerative Models

### 2.1. Neutrophils

Circulating neutrophils act as front-line soldiers of the innate immune system, which are rapidly recruited to inflammatory sites of injury or infection by damage-associated molecular patterns (DAMPs), alarmins, cytokines, and chemokines. As these granulocytes are short-lived, the bone marrow continuously produces neutrophils from hematopoietic progenitors to maintain homeostatic levels for a ready pool of neutrophils during steady-state conditions. Once mobilized to the wound or site of infection, neutrophils ingest cellular debris and invading pathogens by phagocytosis. These leukocytes also release their granular enzymes such as myeloperoxidase and matrix metalloproteases (MMPs) during degranulation for additional antimicrobial and tissue repair/remodeling activity, respectively [60,61,158]. Another critical host defense mechanism provided by neutrophils derives from their formation of neutrophil extracellular traps (NETs), which are composed of chromatin filaments with granular proteins that can obstruct and bind pathogens [158]. In addition to their antimicrobial functions, neutrophils also release various cytokines and extracellular vesicles (EVs) to further modulate the immune response [60,61,158].

After MI in humans, several groups reported a correlation between elevated peripheral blood neutrophil counts (neutrophilia) and increased infarct size, heart failure, and death [49,50,51,52,53,54,55,56]. Whether these neutrophils enter the infarcted myocardium to elicit further damage remains unknown, due to the ethical limitations to human investigations. In non-regenerative adult murine models of MI, neutrophils (Ly6G+) quickly infiltrate the damaged myocardium from day 1 post-injury, with cell numbers peaking at day 3 with permanent ligation and at day 1 with reperfusion. With permanent ligation, neutrophils continued to accumulate and persist from day 1 to day 14, while reperfusion led to a decline in neutrophils from day 3 to day 7 [57,58]. Experimental evidence suggests both detrimental and beneficial aspects of neutrophil infiltration to the infarcted region. Recruited neutrophils perpetuate further damage to the ischemic region with the release of reactive oxygen species (ROS) and proteolytic enzymes which contribute to cardiomyocyte (CM) death and adverse cardiac remodeling [59,60,61]. Neutrophils also support cardiac repair by removing dead cells/debris, promoting angiogenesis, and resolving inflammation [60]. Antibody-mediated (anti-Ly6G) depletion of neutrophils leads to limited cardiac function, enhanced fibrosis, and heart failure development [62]. 

Similar to non-regenerative adults, neonatal mice quickly mobilize neutrophils to the damaged myocardium, with neutrophil numbers peaking at day 1 after injury in both regenerative P1 (1 day postpartum) neonates and non-regenerative P14 (14 days postpartum) juveniles. Both P1 and P14 mice experienced a dramatic decline in neutrophil numbers from day 1 to day 4 after injury, with numbers returning to non-injured levels by day 7 post-injury [63,64]. This is in stark contrast to non-regenerative adult mice, which experience persistent, elevated neutrophil levels at day 14 after injury with permanent ligation and at day 7 with reperfusion [57]. P1 neonatal mice also have significantly lowered neutrophil recruitment after cardiac injury in comparison to non-regenerating adult mice. In contrast to P14 juvenile mice, P1 neonatal mice had significantly elevated steady-state neutrophil levels before injury and at days 7 and 12 post-injury, with diminished neutrophil levels at days 1 and 4 post-injury [63,64]. The regenerative zebrafish model system also demonstrated rapid neutrophil recruitment within 6 h of cryoinjury, with numbers peaking at 1 day after injury. However, after 1 day post-injury (dpi), neutrophil numbers quickly dropped from 3 to 7 dpi and returned to basal levels by 14 dpi [65,66,67,68]. Delayed neutrophil clearance in zebrafish, through depletion of macrophages by clodronate liposomes or treatment with a CXCR1/2 inhibitor, led to an extensive inflammatory period, scar retention, and limited cardiomyocyte renewal [65,67]. Moreover, neutrophil retention inhibited cardiac regeneration despite enhanced revascularization [67], further suggesting the critical nature of the timely recruitment and removal of neutrophils for effective heart regeneration in zebrafish. 

Although both regenerative and non-regenerative systems display swift neutrophil deployment to injured hearts, differences in the timing of neutrophil retention/resolution described above and cell subtypes likely contributes to repair or regeneration outcomes. Quick neutrophil recruitment and resolution likely contribute to regenerative mechanisms, whereas lengthy neutrophil retention and elevated neutrophil numbers in non-regenerative systems hinder the repair process. However, neutrophils still require further characterization in regenerative model systems for comparison with their non-regenerative counterparts. Currently, the requirement for timely neutrophil infiltration and resolution in regenerative neonatal mice has not been examined with antibody or genetic neutrophil depletion models. In addition, further analyses into cardiac neutrophil subtypes in regenerative neonatal mice and zebrafish models, through single-cell RNA-seq (scRNA-seq) for their comparison to non-regenerating juvenile and adult mice, will reveal critical similarities and differences between these models. Future investigations to delineate neutrophil subtypes through scRNA-seq analyses in regenerative systems for comparison with the recently identified murine pro-inflammatory N1 and anti-inflammatory N2 subtypes [159], as well as the generation of specific reporters for these subtypes, are needed. Further, the potential regulation of macrophage phenotypes or other immune cells by neutrophils during cardiac regeneration can only be examined with the creation of newly developed genetic tools that enable specific and temporal neutrophil manipulation. For further information on neutrophils and NETs in myocardial infarction, see the following reviews [61,158,159].

### 2.2. Monocytes/Macrophages

As professional phagocytes of the innate immune system, macrophages perform several critical functions in response to injury or infection. Damage signals rapidly recruit inflammatory monocytes (derived from hematopoietic progenitors) from bone marrow or splenic reservoirs to the wound where they differentiate into macrophages [69,160,161,162]. Mobilized non-tissue-resident macrophages implement several vital functions: they act as professional phagocytes for the removal of pathogens, cellular debris, and dead cells, and neutrophil clearance; present antigens to T cells for an adaptive immune response; and secrete various factors such as proteolytic enzymes and pro-inflammatory cytokines [163]. Tissue-resident macrophages, including those patrolling cardiac tissues, develop from yolk sac and fetal monocyte progenitors [164,165,166]. During steady-state conditions, resident macrophages sustain their local populations through proliferation and only rely on peripheral monocytes for expansion when homeostatic imbalances occur, like with injury or aging [75,77,165,166,167,168]. Resident macrophages patrol their local tissue for pathogens and dead or senescent cells to maintain homeostatic conditions during steady-state conditions [163]. 

After MI in humans, several clinical reports observed an association between elevated pro-inflammatory classical monocyte levels in patient peripheral blood and adverse myocardial recovery [70,71,72,73,74]. Moreover, the monocytes mobilized from bone marrow and splenic reservoirs infiltrated cardiac tissues, as autopsy cases revealed penetration of monocytes (CD14+) to the infarct border region during the inflammatory phase (12 h–5 days post-MI) and invasion of the infarct region during the proliferative phase (5–14 days post-MI) [69]. These recruited monocytes differentiate into macrophages (CD68+), which occur throughout the human heart and are elevated in patients with acute myocardial infarction (AMI) [127,131,168,169]. In adult murine hearts, macrophages form the predominant leukocyte cell type under homeostatic conditions and infiltrate the infarct and border region at 7 days post-injury [57,170]. Further, biphasic recruitment of classical inflammatory Ly6C^hi^ monocytes and macrophages predominates in the early phase (days 1–3 post-injury), while anti-inflammatory reparative Ly6C^lo^ monocytes and macrophages prevail in the later phase from day 5 post-injury and onward [57,76]. Although both pro-inflammatory and anti-inflammatory macrophages decline in number 7 days after injury, both phenotypes persist in the damaged myocardium at 14 days post-injury in elevated numbers in comparison to sham injury controls [57]. Generally, depletion of monocytes/macrophages with clodronate liposome injections after injury in adult mice led to incomplete clearance of necrotic cells, reduced angiogenesis and collagen deposition, induced cardiac rupture, and increased mortality [82]. Specifically, depletion of early-phase monocytes/macrophages with clodronate liposomes led to larger areas of necrotic tissue, cellular debris, and enhanced neutrophil numbers, whereas depletion of later-phase monocyte/macrophages inhibited collagen deposition and granular tissue formation [76]. 

Similar to non-regenerative systems, neonatal mice recruit monocytes in a biphasic manner after coronary ligation with, first, pro-inflammatory Ly6C^hi^ and, then, anti-inflammatory Ly6C^lo^ monocyte infiltration [64]. However, P1 neonatal mice recruit and retain higher levels of monocytes and macrophages throughout the myocardium in comparison to non-regenerating P14 juvenile mice [64]. Analogous to adult and neonatal mammals, zebrafish macrophages form the predominant leukocyte population in injured hearts, with quick mobilization and accumulation observed within 6 h of cryoinjury through 7 dpi. However, zebrafish macrophages return to basal levels by 14 dpi [66,67,69]. Zebrafish cardiac macrophages also demonstrate a biphasic recruitment of distinctive macrophage activation states, with early (1–3 dpi) pro-inflammatory (TNFα+) and later (3–7 dpi) anti-inflammatory (TNFα-) subpopulations [68,83]. The recruited macrophages localize throughout the atrium and ventricle, adjacent to the epicardium and injury site [169]. 

Functional studies with neonatal mice and zebrafish revealed the requirement for macrophages in a cardiac regenerative landscape. Neonates depleted of monocytes/macrophages by clodronate liposome injections or with diptheria toxin (*CD11b^NTR^*) demonstrated abolished heart regeneration capabilities, scar formation, adverse cardiac remodeling, and poor angiogenesis [64,65,171]. Further, transcriptional profiling of monocytes/macrophages isolated from P1 regenerative neonates revealed a pro-regenerative phenotype with significant upregulation of angiogenic genes in comparison to their non-regenerative P14 juvenile counterparts after cardiac injury [64]. Similar to mammals, zebrafish depleted of monocytes/macrophages (clodronate liposomes) or their recruitment delayed with drug treatment (PLX3397, GM6001) experienced heart regeneration inhibition with impairment of CM proliferation, neutrophil clearance, scar resolution, and revascularization [66,67,69,85]. Specifically, depletion at early time points resulted in impaired collagen deposition, whereas depletion of later recruited macrophages had enhanced collagen levels, suggesting early macrophages contribute to scar formation, while later macrophages support scar resolution [68]. It should be noted that these depletion methods are not specific to macrophages, as clodronate liposomes may remove other phagocytic cells such as dendritic cells, and *CD11b* is also expressed in other myeloid cells such as neutrophils or eosinophils [172,173]. Collectively, these results reveal similarities in the biphasic monocyte/macrophage recruitment and their function in neutrophil removal, collagen deposition, and angiogenesis in both regenerative and non-regenerative systems. However, in non-regenerative systems, macrophages persist at the injury site for weeks, while in regenerative systems, they quickly return to basal levels and support CM proliferation and scar resolution for cardiac regeneration. 

Mammalian heart macrophages are also organized into diverse macrophage subpopulations, with reparative tissue-resident (CCR2-) and inflammatory monocyte-derived (CCR2+) groups [75]. The monocyte-derived macrophages likely replace the tissue-resident macrophages, which die in response to ischemic injury [64,167,168]. Embryonically derived tissue-resident macrophages (CCR2-) possess cardioprotective functions, while monocyte-derived macrophages (CCR2+) promote adverse ventricular remodeling. Moreover, the recruited monocyte-derived macrophages persist in the remote myocardium for months after injury in non-regenerative systems for persistent tissue inflammation [77,78,79,80,81]. Furthermore, heart failure patients with left ventricular remodeling have an accumulation of inflammatory monocyte-derived (CCR2+) macrophages, suggesting targeting of this macrophage population may limit adverse cardiac events [75]. 

Differences in cardiac regenerative capabilities between adult and neonatal mice can also be attributed to these different macrophage populations residing in the heart after cardiac injury. Adult mice lose their tissue-resident reparative macrophages (CCR2-) after injury and replace this population with inflammatory CCR2+ monocyte-derived macrophages [64,167,168]. Conversely, neonatal mice do not recruit CCR2+ monocytes after injury and instead expand their resident reparative CCR2- macrophage population [63]. Administration of a CCR2 inhibitor after cardiac injury, which prevents monocyte recruitment to the adult heart, led to preservation of the tissue-resident CCR2- macrophage population, limited inflammation, and elevated angiogenesis [63]. Moreover, transplantation of heart injury macrophages, derived from regenerative neonates, into adult mice with cardiac injury promoted cardiac repair processes with enhanced CM proliferation, a smaller infarct size, and improved cardiac function [171,174].

Whether zebrafish macrophages display different activation states dependent on their origin (tissue-resident vs. monocyte-derived) as previously described in mammals requires further elucidation. It is likely that tissue-resident cardiac macrophages also support regeneration, as observed with zebrafish tail fin regeneration [175,176]. However, reparative *wt1b*+ macrophages, derived from the kidney marrow (equivalent to the bone marrow in mammals), accumulate in the regenerating heart and support CM proliferation in contrast to the detrimental non-resident macrophages in adult mammals [85]. Additionally, both tissue- and monocyte-derived macrophages have long been categorized into classical inflammatory and alternatively activated reparative states based on their inflammatory status [177]. However, this outdated classification system greatly oversimplifies macrophage phenotypes [178], as macrophages are a more complex and heterogenous cell population than previously reported. A recent study demonstrated nearly 300 diverse macrophage transcriptomes in response to various stimuli [179], and other studies indicate novel functions for these heterogenous cells in angiogenesis, collagen deposition, and electrical conduction [173,180,181]. Although temporal differences in macrophage retention are clearly observed with lengthy macrophage persistence in non-regenerative systems (weeks to months) and macrophage resolution by 2 weeks in regenerative systems, these macrophage subtypes require further elucidation in both systems. Therefore, future studies demand specific markers to classify and analyze this heterogenous population in both regenerative and non-regenerative systems. As recent reports demonstrated that the widely used zebrafish macrophage marker *mpeg1.1* labels a subpopulation of B cells and natural killer-like cells, future zebrafish studies also require a more specific macrophage reporter line [182,183]. Further, additional reporter lines to specifically label and manipulate macrophage subtypes will need to be developed in both regenerative and non-regenerative systems to classify their functional roles. For additional information on monocyte/macrophage roles in myocardial infarction, see the following reviews [163,180,181,184].

### 2.3. T Lymphocytes

As part of the adaptive immune response, T lymphocyte subsets perform diverse roles, including recognizing various antigens from pathogens to tumors and maintaining homeostasis, tolerance, and immunological memory. T cells develop from hematopoietic progenitors in the bone marrow before maturation into helper (CD4+), cytotoxic (CD8+), or regulatory (Treg) subsets in the thymus [185]. CD4+ helper T cells release various cytokines to regulate leukocyte activity, and Treg cells suppress the immune system and prevent self-reactivity, while CD8+ cytotoxic T cells directly kill target cells. Cytokine profiles further subdivide helper T cells into inflammatory IFN-γ- and TNFα-producing Th1 cells; IL-4-, IL-5-, and IL-13-generating Th2 cells which support humoral immunity and parasite defense; IL-17-producing Th17 cells; IL-22-releasing Th22 cells; and IL-9-generating Th9 cells [186]. Antigen-presenting cells (APCs) such as monocytes, macrophages, dendritic cells, and B cells collect, process, and present antigens to naïve T cells for their activation. Further, for an effective immune response to occur, T-cell priming requires associations between co-stimulatory molecules on the surface of APCs and T cells in addition to the interaction between the antigen-bound major histocompatibility complex (MHC) of an APC and the T-cell receptor (TCR) [187]. Once activated, clonal expansion ensues for mass production of short-lived effector T cells (either helper, cytotoxic, or regulatory), capable of mediating a targeted immune response. Although most effector cells undergo apoptosis, some persist as long-lived memory T cells, which circulate in the periphery and rapidly expand into large numbers of effector T cells upon additional encounters with their specific antigen. Most T cells localize to lymphoid tissues such as the bone marrow, spleen, thymus, and lymph nodes. However, T cells occur throughout the body in all major organs and tissues and circulate in the peripheral blood [185]. Moreover, tissue-resident memory T cells occur in the myocardium, where they provide rapid protection against pathogens [188].

Clinical studies examining T-cell responses after myocardial infarction (MI) in humans are limited to either analyzing patient peripheral blood subsets or immunostaining myocardial tissue. Immediately after MI in humans, there is a significant decrease in the number of CD4+ helper and CD8+ cytotoxic T cells in the periphery, while 4–6 days later, the number of Tregs is enhanced [86,87,88]. However, other reports conducted on peripheral blood from acute coronary syndrome (ACS) patients demonstrated elevated levels of Th1 and Th17 CD4+ cells with concurrent decreases in Treg cells [89,90,91,92]. The observed differences in the T lymphocyte count in the periphery likely arise from different methodologies in various studies. Moreover, analysis of circulating T cells only represents a small fraction of the adaptive immune response to heart injury and fails to demonstrate leukocyte infiltration to the damaged myocardium. During steady-state conditions, the adult murine heart maintains low levels of T cells, while coronary arterial ligation models of MI lead to a nearly 10-fold enhancement of cardiac T-cell numbers, suggesting robust recruitment to the infarcted region [170,189].

Analysis of the myocardium from human autopsies revealed penetration of CD3+ T lymphocytes in the remote and peri-infarct region of ischemic hearts, including coronary arterial walls [190,191]. Moreover, patients with acute MI had both CD4+ helper cells and Foxp3+ Treg cells within the infarcted region [108]. Patient biopsies also demonstrated an enrichment of CD4+ Th1 and CD8+ T cells in failing hearts, as well as their potential activation with co-localization of T cells and APCs such as macrophages and dendritic cells [95,96]. In adult murine MI models, CD4+, CD8+, and γδ T cells mobilized to the injured myocardium from day 1, peaking at day 7 with permanent occlusion and at day 3 with reperfusion. Total T-cell numbers then lowered at day 14 with permanent ligation and at day 7 with reperfusion, but remained significantly elevated [57,88,99,100,101,102]. The majority of CD4+ T cells that localized to the damaged hearts were Th1 and Treg subtypes. However, both Th2 and Th17 CD4+ subsets also infiltrated the injured murine heart [57]. 

Divergent reports implicate CD4 T-cell subsets as either exacerbating or promoting cardiac repair in non-regenerative adult mammals after injury. Clinical studies investigating the level of IL-17 (produced from Th17 cells) in the circulation provided conflicting results, with some reports showing an association between enhanced IL-17 levels and poor patient prognosis, while another study found lower IL-17 levels were linked with adverse cardiac events [92,93,192]. CD4+ T cells aggravate cardiac injury in adult mice, as antibody-mediated (anti-CD4) depletion of CD4+ T cells resulted in smaller infarcts after injury. Further, mice deficient in mature lymphocytes (*Rag1* knockout) possessed smaller cardiac scars than control wild-type mice. However, adoptive transfer of CD4+ T cells into *Rag1* knockout mice eliminated their previously observed cardioprotective benefits [99]. Additionally, CD4+ T cells persist, expand several weeks after cardiac injury, and contribute to heart failure. Antibody-mediated depletion of CD4+ T cells at 4 weeks post-injury limited their infiltration and prevented ventricular dysfunction [102]. In opposition to these reports, CD4 knockout mice displayed adverse cardiac function with enhanced recruitment of Ly6C^hi^ monocytes to the infarct, altered collagen deposition, and limited neovascularization [88]. Additionally, T-cell activation is required for cardiac repair, as ablation of professional antigen-presenting cells such as dendritic cells (CD11c+) leads to adverse cardiac remodeling [139]. Moreover, CD4+ T cells activated by the myosin heavy chain alpha (MYHCA) cardiac antigen develop a Treg cardioprotective phenotype with the expression of reparative genes that support collagen deposition for tissue repair [108]. Differences observed in the functional role of CD4+ T cells likely derive from the depletion methods utilized, as both knockout and antibody-mediated loss of CD4+ T cells broadly target both conventional helper and Treg cells. Further examination of conventional helper T cells and their subtypes in repair after injury is currently lacking, but several studies have exclusively focused on the role of Tregs.

Lower levels of Tregs in heart failure patients correlate with left ventricular remodeling, cardiac dysfunction, and lowered survival [97,98]. Although some studies suggested that antibody-mediated (anti-CD25) Treg depletion in adult ischemic mice had no significant effect on infarct size, cardiac function, or remodeling [109,193], other studies revealed its role in supporting wound recovery [100,110,111,112,113,114,115]. Antibody- or diptheria toxin (Foxp3-NTR)-mediated Treg depletion and Treg recruitment deficiencies significantly enhanced the mobilization of pro-inflammatory leukocytes such as neutrophils, Ly6C^hi^ monocytes, CD4+ and CD8+ T cells, and classically polarized macrophages to the infarcted region [100,109,110,111]. Further, Treg loss reduced cardiac function with lowered collagen deposition and elevated adverse remodeling [100,109,110,111]. Moreover, adoptive transfer of Tregs into the infarcted region significantly reduced cardiac injury by limiting the inflammatory response, lowering the infarct size, increasing CM proliferation, and alleviating adverse remodeling [112,113,114,115,193]. The largely beneficial effects of Tregs on cardiac recovery arise from their suppression of inflammatory leukocytes to support the transition to a reparative environment.

CD8+ and γδ T cells predominantly exhibit detrimental effects on cardiac recovery after MI. AMI patients with elevated levels of CD8+ CD28+ T cells had larger infarcts and lowered ventricular function, suggesting a contribution of this subtype to myocardial damage [93]. Another cytotoxic T-cell subtype, CD8+ CD57+, may also exacerbate tissue damage, as higher levels were positively correlated with increased mortality 6 months after AMI [94]. Depletion of cytotoxic CD8+ T cells in adult mice through a CD8-targeted antibody or genetic strains deficient in functional CD8+ cells (CD8a^tm1mak^) demonstrated enhanced cardiac function, with decreased CM apoptosis and a lowered inflammatory response with macrophage polarization towards a reparative anti-inflammatory phenotype [103,104,105]. Although CD8+ T-cell activation contributes to CM-specific toxicity [194] and adverse ventricular remodeling [103], CD8+ T-cell loss leads to compensatory hypertrophy, enhanced cardiac rupture, and scar formation [104,105]. Further, a subtype of CD8+ T cells, positive for angiotensin 2 receptor (AT2R+) discovered in rat models of MI, secretes IL-10 in response to angiotensin 2 and supports cardiac healing, with decreases in infarct size after adoptive transfer of these cells to ischemic hearts [101]. Therefore, future more in-depth analyses on the various cytotoxic T-cell subtypes are required to fully elucidate beneficial and harmful subtypes in rodent models of MI. γδ T cells also infiltrate the infarcted myocardium and release IL-17A, which contributes to the continued infiltration of inflammatory leukocytes, promotes CM death and fibrosis, and supports adverse cardiac remodeling [58,106,107].

In the neonatal murine model of MI, CD4+ and CD8+ T cells mobilized to injured hearts from days 1 to 7 post-injury in both P3 (3 days postpartum) regenerative neonatal and P8 (8 days postpartum) non-regenerative juvenile mice, with numbers peaking at day 7. However, these T cells infiltrated the infarct region in significantly higher numbers in P8 mice in comparison to P3 mice at all time points before and after injury. Although CD8+ T cells returned to basal levels by day 14 post-injury in both P3 and P8 mice, CD4+ T cells remained substantially elevated at day 14 in P8 mice while returning to basal levels in P3 mice [117]. Regarding zebrafish, cryoinjury leads to significant upregulation of genes involved in T-cell proliferation in zebrafish but not in the non-regenerative medaka [60]. Further, after cryoinjury, T cells (*lck*:GFP) mobilize to the wounded area from day 1, peaking at 7 dpi and resolving by 14 dpi [68]. Antibody-mediated (anti-CD4) depletion of CD4+ T cells facilitated cardiac regeneration in P8 mice through alleviation of cardiac fibrosis and enhanced CM proliferation [117]. Furthermore, scRNA-seq revealed a pro-fibrotic CD4+ T-cell subset only in P8 mice, which likely derives from enhanced numbers of Th1 and Th17 cells secreting cytokines that both limit CM proliferation and induce apoptosis of CMs [117]. To date, the roles of CD4+ T cells and their subtypes have not been examined in the regenerative zebrafish model after heart injury. 

Recent studies revealed beneficial roles for Tregs in cardiac regeneration in both neonatal mice and zebrafish systems. Neonatal mice demonstrated an innate preference for Treg production, with nearly 70% of their T-cell precursors differentiating into Tregs, whereas adults generated less than 10% [195]. This bias for Treg differentiation diminished within the first two weeks of life, which corresponds to the loss of cardiac regeneration abilities in neonatal mice, suggesting a functional role for Tregs in cardiac regeneration [13,14,196]. Further, analysis of regenerating P3 mice and non-regenerating P8 mice established Treg cell recruitment within the first week of cryoinjury and significantly enhanced cell numbers in regenerative neonates [116]. Moreover, antibody-mediated (anti-CD25) or diptheria toxin (Foxp3^NTR^) depletion of Tregs resulted in enhanced cardiac fibrosis after injury, while adoptive transfer of Tregs from neonatal or adult mice into T cell-deficient (NOD/SCID) P3 neonates supported cardiac function and regeneration after injury, with elevated CM proliferation and a reduction in cardiac fibrosis [116]. The specific role of Tregs in zebrafish cardiac regeneration has been observed, with the utilization of the *foxp3a* transcription factor to specifically label this T-cell subtype [197]. During steady-state conditions, Tregs predominantly localize to the kidney, with some found in the spleen and thymus, and no Tregs in the heart. In response to heart injury, Tregs mobilize to the ischemic region and peak at 7 dpi. Specific depletion of Treg cells (*fox3pa*:NTR) after heart injury resulted in thinner myocardial walls, a persistent collagenous scar, lowered CM proliferation, and macrophage polarization towards the pro-inflammatory state [118]. 

Overall, non-regenerative studies on both CD4+ and CD8+ T cells revealed conflicting results, with some analyses demonstrating harmful effects and others identifying beneficial subtypes. However, regenerative systems established detrimental roles for CD4+ T cells in cardiac regeneration, with these cells contributing to fibrosis and inhibiting regeneration in juvenile mice. In opposition, CD8+ T cells may have no beneficial role in regenerative systems as CD8+ T-cell depletion had no effect on neonatal regeneration [117]. Although both non-regenerative and regenerative model systems displayed similar T-cell mobilization from day 1 with numbers peaking at day 7 post-injury, differences occurred in T-cell resolution. Non-regenerative adults and juveniles retained elevated T-cell numbers by 14 days post-injury, with CD4+ T cells persisting for weeks or months after injury. In comparison, regenerative neonatal mice and zebrafish returned T cells to basal levels by 14 days post-injury. Critically, both non-regenerative and regenerative systems established vital roles for Tregs in both repair and regeneration, likely through their regulation of other leukocytes to manipulate the inflammatory environment. 

Despite these findings, significant knowledge gaps remain. Various subtypes of CD4+, CD8+, Tregs, and γδ T cells have yet to be identified and analyzed in each model system with depletion methods. Moreover, despite zebrafish containing T-cell subtypes similar to mammals such as CD4+ T cells [192,198], Tregs [199,200,201], and γδ T cells [202], only Tregs have been characterized during heart regeneration. This is due to a lack of specific cell markers and antibodies to label these other cell populations. Therefore, cell-specific markers need to be identified through scRNA-seq analyses to ensure the development of cell-specific genetic tools or mutant lines for the zebrafish system. Further examination of other T-cell subtypes (CD4+, CD8+, and γδ T cells) with both non-regenerating and regenerating systems will elucidate the function of these cells in a cardiac repair or regenerative environment. For further information on T-cell roles in myocardial infarction, see reviews [194,203,204].

## 3. Leukocytes Currently Only Examined in Non-Regenerative Models

### 3.1. Eosinophils 

Eosinophils differentiate from bone marrow progenitors before their release into the bloodstream or tissues for immune surveillance [196,205]. Despite their limited numbers in the peripheral blood during steady-state conditions (1–3%), these multipurpose cells perform key functions in diverse biological processes [119]. As a granulocytic leukocyte, a well-established eosinophil function includes releasing their toxic granule components, such as RNases, for host defense against viral, bacterial, and helminth pathogens [205]. Other granular contents include numerous cytokines, chemokines, growth factors, bioactive lipids, and enzymes [196]. These granules contribute to other eosinophil functions such as facilitating both innate and adaptive immune responses; supporting tissue and metabolic homeostasis; and tissue remodeling and fibrosis [199]. 

In the human heart, eosinophils mobilize and accumulate after ischemic injury [206]. Previous studies revealed associations between lowered eosinophil levels after MI and an increased risk for myocardial damage, major adverse cardiac events (MACE), and death [119,120,121,122,123,124]. Similarly, a more recent study demonstrated enhanced eosinophil levels in the peripheral blood and cardiac infarct, which provided cardioprotective functions after MI to limit CM death, cardiac fibrosis, and inflammatory cell accumulation [125]. In contrast, one report observed an initial protection from elevated peripheral eosinophils at 6 months post-reperfusion but an increased risk of death at later time points [123]. Therefore, the precise role of eosinophils after MI in non-regenerative systems and whether they support cardiac repair remain unclear and require further study in animal models. In an adult mouse model of MI, using left anterior descending coronary artery ligation, eosinophils increased from days 1 to 4 post-injury, peaking at day 4 and decreasing at day 7. Importantly, genetic (ΔdblGATA) or antibody-mediated eosinophil depletion led to more severe cardiac dysfunction after injury, including a larger cardiac infarct size as well as increased cell death and collagen deposition. Moreover, treatment with IL-4 therapy in eosinophil-deficient mice rescued adverse cardiac remodeling phenotypes, implicating eosinophil IL-4 secretion in tissue homeostasis [124]. Other animal model studies also demonstrated an intriguing role for IL-4-secreting eosinophils in tissue repair or regeneration after liver or skeletal muscle injury [200,207].

Despite the renewed interest in eosinophil function after MI due to their involvement in tissue remodeling and homeostasis [199], their role in heart regeneration has not been characterized in zebrafish or neonatal mice. Zebrafish eosinophils morphologically resemble mammalian eosinophils (round cell shape with segmented nuclei) and express similar genes. A recent zebrafish study examined the inflammatory response to cardiac injury, by utilizing the transgenic line Tg(*gata2a:eGFP*) to observe eosinophil mobilization after cryoinjury [68,201]. Without injury, there were little to no eosinophils observed in the zebrafish heart. However, after cryoinjury, there was a significant number of eosinophils at 6 h post-injury as well as at 7, 14, and 21 days post-injury [68]. Although definitive answers to questions regarding if and how eosinophils contribute to the neonatal or zebrafish regenerative model systems are not known, the rapid and sustained infiltration of eosinophils to injured cardiac tissue suggests an integral function for these cells in regeneration. In future analyses, genetic or antibody-mediated depletion of eosinophils can be applied in these regenerative systems to determine whether eosinophils support cardiac regeneration.

### 3.2. Basophils 

The bone marrow serves as the primary source of basophils, which quickly mobilize to inflammatory regions and exert their function by releasing effector molecules such as cytokines, enzymes, histamine, and bioactive lipids. Basophils are predominantly known for their roles in allergic reactions and protection against helminth pathogens [208]. Recent reports suggest other functional roles for these effector cells, such as regulating macrophage development and function [203]; defense against bacterial infections [204]; and fibroblast activation and organ remodeling [209]. 

Interestingly, a recent clinical study found that both low and high basophil counts in patient peripheral blood after MI were associated with higher mortality rates [122]. Another report also examined basophil cell counts in patients after MI. This study demonstrated a gradual increase in basophil numbers after heart attack, with a peak at 96 h post-MI. Moreover, lower basophil numbers in the peripheral blood during the first week post-MI correlated with a larger scar size and adverse cardiac events one year after MI [126]. Adult mouse models of MI, using left anterior descending artery (LAD) ligation, demonstrated elevated basophil numbers with recruitment from day 1 post-injury, peaking between days 3 and 7 and returning to baseline by day 14 [126]. Moreover, both antibody-mediated (MAR-1) and genetic (Mcpt8-Cre transgenic strain) basophil depletion led to severe cardiac dysfunction after injury, such as larger end-diastolic and end-systolic left ventricular volumes, enhanced heart weight-to-body weight ratios, and a reduced scar thickness [126]. 

Based on the previous literature that demonstrated the role of basophils in macrophage development/function, monocyte phenotypes, and organ remodeling [126,210,211], basophils likely play critical regulatory roles in the inflammatory response in regenerative systems. At present, basophils have not been characterized in cardiac regenerative model systems such as neonatal mice or zebrafish. Basophils have yet to be identified or examined in zebrafish, but they have been identified in other teleost species [201,212]. As basophils are evolutionarily conserved in vertebrates [213], it is likely that zebrafish do have basophils, but the limited number and short lifespan of these cells have contributed to a delay in their discovery. As basophil levels after cardiac injury in non-regenerative models correlated with heart recovery, it will be of particular interest to examine basophil localization to the cardiac injury area in regenerative systems, whether they also support a reparative environment, and the effect of basophils on other recruited cardiac leukocytes, particularly monocytes/macrophages.

### 3.3. Dendritic Cells 

Dendritic cells (DCs) consist of heterogenous cell populations responsible for connecting the innate and the adaptive immunity, inducing and regulating immune responses, and maintaining self-tolerance. DC precursors develop from hematopoietic stem cell progenitors in the bone marrow and give rise to immature DCs that circulate and conduct immune surveillance in tissues and the peripheral blood [214]. Once activated, these professional antigen-presenting cells migrate to lymphoid organs such as the spleen or lymph nodes, where they interact with B and T cells to initiate an appropriate immune response. Classification of DCs includes steady-state DCs, inflammatory DCs, and Langerhans cells [214]. Steady-state DCs are further divided into conventional/classical (cDCs), myeloid (mDCs), and plasmacytoid (pDCs). cDC1s have roles in cross-presentation; activating CD4+ T cells, CD8+ T cells, and natural kill cells; and recruiting neutrophils. cDC2s present antigens to activate CD4+ T cells and induce Treg, Th1, and Th17 cells. pDCs are well known for producing large quantities of type I interferon (IFN-I) and have roles in initiating antiviral immune responses and inducing tolerance [214]. Inflammatory DCs differentiate from monocyte precursors in response to infection or injury and migrate to lymphoid organs to present their antigens to T cells. Langerhans cells (LCs) are of embryonic origin and restricted to the epidermis. As with other DC subtypes, LCs migrate to lymphoid tissues upon activation to present their antigen to activate T cells and induce an immune response [214]. 

In patient peripheral blood after AMI, several clinical reports identified a significant decrease in the number of circulating DCs or DC precursors in patient peripheral blood after AMI [127,128,129,131]. The limited number of peripheral blood DCs after MI may result from their recruitment to the damaged heart, where they likely activate T cells for an immune response. The amount of activated DCs, as evidenced by increased levels of DCs with maturation markers CD40+ and CD83+, also increased after AMI, suggesting their activation due to necrotic cells or damage signals [129]. In adult rodent models of MI, DCs localized to the infarcted myocardium and border regions, accumulating from day 1 and peaking at 7 days post-injury with permanent ligation and at day 3 with reperfusion. Although DC numbers decline at day 14 with permanent ligation and at day 7 with reperfusion, these cells are still significantly elevated in comparison to sham controls [57,133]. Further, all DC subsets mobilized, permeated, and matured within the infarcted murine myocardium [95,137]. 

Notably, several animal model studies of MI suggest DC infiltration hinders heart repair. Enhanced numbers of mature DCs at the infarct region exacerbated ventricular remodeling, whereas diminished DC infiltration prevented adverse remodeling [134]. Genetic knockout of *IRAK-4*, a component necessary for DC mobilization from the bone marrow, demonstrated cardiac benefits after injury with improved cardiac function, a smaller infarct size, and lower fibrosis [135]. Additionally, specific antibody depletion of pDCs (anti-PDC1-A) or blocking factors responsible for IFN-1 production showed smaller infarcts after injury, suggesting that the large amounts of IFN-1 released from infiltrated pDCs contribute to harmful inflammatory responses after ischemic injury [138]. Further, angiotensin-converting enzyme inhibitors (ACEI) such as lisinopril suppress cardiac remodeling after injury by limiting the maturation and recruitment of DCs from the spleen [215,216]. 

Clinical reports and adult murine studies also demonstrated beneficial roles for DCs in tissue repair after heart damage. One study found that lower DC counts correlated with enhanced macrophage numbers within infarcts, limited reparative fibrosis, and cardiac rupture [132]. Specifically, genetic DC (CD11c+) depletion in mice revealed diminished ventricular function and adverse remodeling after injury [139]. Another study implemented the adoptive transfer of splenic mononuclear cells (MNCs) from DC-depleted (CD11c+) or control mice into splenoctomized mice after injury. Mice transplanted with control MNCs had larger capillary densities, smaller infarcts, and less severe cardiac remodeling and dysfunction, in comparison to mice transplanted with DC-depleted MNCs after cardiac injury [140]. Adoptive transfer of tolerogenic DCs, conditioned with IL-37 or IL-38 and troponin-I, lowered myocardial fibrosis and the permeation of inflammatory leukocytes to the infarcted region, leading to improved cardiac function [217,218]. Additionally, injection of infarct-primed tolerogenic DCs leads to an activation of regulatory T cells, a shift from an inflammatory to reparative macrophage environment, diminished adverse cardiac remodeling, and neo-angiogenesis [210]. The beneficial effects of treatment with tolerogenic DCs after cardiac injury likely result, in part, from the large amounts of exosomes released from DCs in response to injury. As with tolerogenic DCs, injection of injury-primed DC exosomes (DEXs) improves cardiac function, enhances angiogenesis, causes a shift to a reparative macrophage environment, limits apoptosis of CMs, and reduces infarct size [211,219,220].

Discrepancies in the functional role of DCs after cardiac injury in non-regenerative systems are likely due to the examination of various DC subtypes in isolation vs. the total DC population. As these cells are heterogenous cell populations, the use of specific genetic markers to characterize each subtype after injury in adult murine models is required to clearly elucidate the role of each subtype in cardiac repair. 

Previous studies in non-regenerative systems implicate DCs as regulators of the cardiac immune environment through their interaction with various T-cell subtypes. For example, recruited DCs presenting cardiac antigens to cytotoxic CD8+ T cells lead to further cardiac damage [95,137], while tolerogenic DCs activate Tregs to diminish the inflammatory macrophage response [210]. Based on these reports, DCs most likely manipulate the inflammatory environment in regenerative systems to support quick inflammation resolution for regenerative mechanisms. However, DCs have yet to be examined in cardiac regenerative systems such as neonatal mice or zebrafish, and thus it remains unclear whether DCs support regeneration. Currently, there are no available reporters or antibodies specific for zebrafish DCs to examine their role during heart regeneration. Previous identification of DCs in zebrafish relied on their morphology and high affinity for lectin peanut agglutinin (PNA^high^) or their isolation with other mononuclear phagocytes from the double transgenic reporter *mhc2dab:GFP;cd45:DsRed* [221,222]. As zebrafish DCs also demonstrate phagocytosis capabilities and T-cell activation and express genes related to mammalian DC function and antigen presentation, these cells likely play critical regulatory roles in the regenerative response [221,223]. The identification of specific markers for zebrafish dendritic cells, likely through scRNA-seq studies, will enable the characterization of these cells during zebrafish heart homeostasis and regeneration. Further, sequencing analyses of zebrafish and neonatal mouse DCs during regeneration will enable critical comparisons with non-regenerative model systems. 

### 3.4. Natural Killer Cells 

Natural killer (NK) cells, which comprise part of the early innate immune response, directly release cytotoxic perforin or granzyme granules to lyse viral-infected or tumor cells [224,225]. Moreover, these early effector cells also regulate the inflammatory response through lysis of activated immune cells and by releasing various cytokines and chemokines [226]. The combined effect of both activating and inhibitory NK cell surface receptors interacting with their target cells determines whether cell lysis occurs [227]. Recent reports also indicate an immune memory-like ability in NK cells [228]. 

After human AMI, several reports indicated either enhanced or diminished levels of circulating NK cells in the peripheral blood [141,142,143]. Despite differences in NK cell numbers, NK cells infiltrated the infarcted region of AMI patients, suggesting recruitment of NK cells from the periphery [142]. Multiple studies also demonstrated NK cell infiltration into infarcted adult mouse myocardia, accumulating from day 1 and peaking at day 7 post-injury with permanent ligation and at day 3 in ischemia–reperfusion studies. These cells decline significantly in number but remain at elevated levels above sham controls at day 14 with permanent ligation and at day 7 with reperfusion [58,59,146]. 

Several clinical reports revealed significant reductions in NK cell cytotoxicity after ischemic injury, suggesting defective NK cell functionalities after MI [143,144,145]. Moreover, a recent microarray analysis revealed downregulation of both activating and inhibiting NK cell receptors (NKR) in response to AMI in comparison with healthy controls [229]. Interestingly, Ortega-Rodriquez et al. observed an elevation in circulating IL-10+ NK cells at 72 h after MI and enhanced functional recovery in patients with reduced IL-10+ NKs at 3 months post-MI [143]. These results led the authors to suggest IL-10 production by NK cells may have a role in regulating inflammation to limit adverse cardiac remodeling. Additionally, c-kit signaling after cardiac injury mobilizes bone marrow-derived NK cells towards the infarct, where they support cardiac remodeling and function [146]. Interleukin-2 (IL-2)-activated NK cells promote angiogenesis and reduce fibrosis of the infarcted myocardium [147,148]. In contrast, antibody-mediated depletion (anti-NK1.1) of NK cells at 24 h before injury significantly reduced the infarct size and limited adverse cardiac remodeling, possibly by reducing neutrophil infiltration [149]. Future investigations regarding whether ischemia leads to NK cell dysfunction in adult animal models, the identification of NK cell subtypes after cardiac injury with scRNA-seq, and which subtypes support or hinder cardiac repair will further delineate NK function in non-regenerative systems.

NK cells have yet to be characterized in the regenerative neonatal or zebrafish model systems. Based on the available literature on non-regenerative systems, specific NK cell subtypes may support cardiac regeneration by limiting neutrophil mobilization to regulate inflammation and promote angiogenesis. Currently, there is no specific genetic reporter or antibody available to study NK cells in zebrafish, hindering the examination of their functional role during heart regeneration in this model system. Despite these limitations, multiple reports indicate NK-like cells exist in zebrafish. These studies revealed a NK-like cell population expressing NK-lysin genes, various cytokines/receptors, and members of the perforin and granzyme secretory pathway [230,231,232,233,234]. Further in-depth examination of single-cell transcriptomic reports will identify relevant candidate genes for screening analyses. This may lead to the eventual identification of a specific NK cell reporter line in zebrafish for future studies. Prospective analyses in both neonatal mice and zebrafish regenerative systems will reveal critical NK cell subtypes through scRNA-seq. For further information on NK cell roles in myocardial infarction, see the following review [235].

### 3.5. B Lymphocytes

B cells form the predominant components of the humoral immune response due to their generation of antigen-specific antibodies. However, B lymphocytes also play significant roles in innate and adaptive immune cell regulation through antigen presentation and the release of cytokines and chemokines [236]. B lymphocytes contain two major subtypes: the prenatally produced innate-like B1 cells that persist from self-renewal, and the postnatally generated traditional B2 cells. B1 cells, which are further divided into B1a and B1b, amass in the peritoneum and produce neutralizing antibodies with low affinity and broad reactivity [236]. Follicular and marginal zone B cells, classified as B2 cells, reside in lymphoid organs, such as the spleen. Both B1 and B2 cells differentiate into antibody-producing cells (long-lived plasma cells or short-lived plasmablasts) upon activation by a specific antigen for their B-cell receptor (BCR) [236]. However, only follicular B cells differentiate into germinal center (GC) B cells and undergo class switching with interactions from helper T cells in the GC of lymphoid organs. These GC B cells can then further differentiate into plasma cells or memory B cells [236]. Marginal zone B cells and B1 cells also produce memory B cells, but their mechanisms remain unclear. Anti-inflammatory B cells, referred to as B-regulatory cells (Bregs), secrete anti-inflammatory molecules such as interleukin-10 (IL-10), interleukin-35 (IL-35), and transforming growth factor β (TGFβ) and induce regulatory T cells for inflammation resolution [236]. Additionally, a subset of B1 cells, referred to as innate response activator (IRA) B cells, express granulocyte-macrophage colony-stimulating factor (GM-CSF) and interleukin-3 (IL-3) and modulate both innate and adaptive immune cell functions [236].

The prevalence of B cells within the human heart and their response to myocardial infarction are far from clear. Autopsies performed on non-cardiac death patients revealed the presence of B lymphocytes (CD19^+^) within the heart [150]. Another report demonstrated a small decrease in circulating B cells after MI but an increase 24 h after reperfusion [86]. Similar to humans, B cells also localize to adult murine hearts during steady-state conditions [170]. In an adult mouse model of MI, B lymphocytes gradually increased in number from day 1 until peaking at day 7 post-injury and decreasing at day 14. However, B cells peaked at 3 days post-injury in an ischemia–reperfusion injury model and lowered in number at day 7 post-injury [57]. 

B cells demonstrated adverse effects on myocardial repair after ischemia-induced injuries in adult mammal studies, while other reports suggest possible beneficial functions for B cells during cardiac repair. Both genetic (Baff receptor) and antibody (anti-CD20)-mediated depletion of B2 lymphocytes limited cardiac injury, blocked adverse LV remodeling, and enhanced cardiac function [152]. Another group observed exacerbated cardiac fibrosis and remodeling after cardiac injury with more enhanced B-cell numbers (CB2-deficient mice) [153]. In addition, the cardioprotective benefits of pirfenidone were recently attributed to lowered levels of cardiac B2 lymphocytes [151]. Furthermore, when examining MI in a B-cell knockout background, the mice showed lowered pro-inflammatory cytokine levels, ventricular remodeling, and cardiac fibrosis [154]. Other animal model studies suggest beneficial roles for B cells. In a rat model of MI, intramyocardial injections of bone marrow-derived B cells reduced apoptosis and improved cardiac function [155]. Cardiac injury in mice led to expansion of an IL-10-producing B1a (CD5^+^) lymphocyte population in the pericardial adipose tissue. These B1a cells also assembled in the infarcted heart during the inflammation resolution period. Knockout of IL-10-producing B cells led to aggravated cardiac injury and impaired cardiac function [156]. Moreover, adoptive transfer of Bregs after injury resulted in a significant enhancement of cardiac function and reduced infarct size and fibrosis [157]. 

In contrast to the above-mentioned studies demonstrating a detrimental or beneficial role of B cells after cardiac injury, a recent study, which identified a novel population of heart-associated B (hB) cells, showed that depletion of hB cells through CXCR5 deficiency or with CXCL13 antibodies had no effect on heart function after injury [237]. Most likely, the differences observed in beneficial and detrimental effects on cardiac repair are due to the examination of different B-cell subtypes. As B lymphocytes are a heterogenous population, the exact role of each cell subtype in cardiac repair can only be elucidated when specific reporters and genetic tools have been developed for each cell subtype. 

Regarding cardiac regeneration models, scant information is available on the function of B cells and their subtypes during heart regeneration. As various B-cell subtypes exacerbate cardiac remodeling through pro-inflammatory cytokine release or support cardiac repair with anti-inflammatory cytokine production in non-regenerative systems, B cells likely have critical regulatory roles in the inflammatory environment in regenerative systems. In a recent single-cell RNA and ATAC sequencing transcriptome in neonatal mice after heart injury, there was an observed increase in B lymphocytes [238]. Similarly, a recent report comparing the response to cryoinjury in the regenerative zebrafish and the non-regenerative medaka showed an elevation in genes associated with B cells in the zebrafish transcriptome after heart injury, but not in the medaka [65]. Moreover, another zebrafish study identified a mixed population of B cells and natural killer-like cells (*mpeg1+ csf1ra-*) that increased over time after cryoinjury [182]. These studies suggest that B cells and their subtypes likely have a role in cardiac regeneration. However, to date, no studies have examined the general role of B cells in a cardiac regenerative environment, despite available reporters. For example, the *IgM1:eGFP* zebrafish B-cell reporter line [239] can be utilized in future studies to study the B-cell temporal response to heart injury and identify B-cell subtypes with scRNA-seq. Only once B lymphocyte subtype markers have been identified and genetic tools are developed can the specific time course of B cells and their subtypes be identified. Further, prospective analyses can analyze various B-cell subtypes during zebrafish and neonatal cardiac regeneration for comparison with cardiac repair in non-regenerative systems. For further information on B-cell roles in myocardial infarction, see reviews [236,240].

## 4. Leukocyte Interactions Examined in Non-Regenerative and Regenerative Models

During tissue repair and regeneration, both innate and adaptive immune cells communicate amongst each other and with other cell types (fibroblasts, CMs, endothelial cells) to orchestrate the immune response. Presently, no differences in immune cell interactions between non-regenerative and regenerative systems are known due to current study limitations. For example, previous clinical studies and both non-regenerative and regenerative animal model systems of MI are often limited to only studying one immune cell type in isolation. Further, there is a significant knowledge gap in regard to the role of several leukocytes (eosinophils, basophils, DCs, NK cells, and B cells) in the zebrafish regenerative system and how they affect other leukocytes. Additionally, information on the role of immune cells during neonatal regeneration is limited to neutrophils, monocytes/macrophages, and T cells, creating further knowledge deficiencies on leukocyte interactions during cardiac regeneration. Here, we briefly discuss leukocyte interactions during cardiac repair. For further information on the interaction of immune cells with resident cardiac tissues and other cell communication during repair and regeneration, please see [241,242,243].

Various immune cells manipulate the inflammatory environment after injury through their interaction with macrophages to regulate their inflammatory state. For example, it is well documented that macrophage clearance of apoptotic neutrophils supports the transition of macrophages towards an anti-inflammatory phenotype [4]. Specifically, antibody-mediated neutrophil depletion in adult mice after cardiac injury resulted in cardiac dysfunction from the inhibition of macrophage polarization towards a reparative phenotype, with an extensive inflammatory phase [62]. Additionally, a proposed mechanism for eosinophils in heart repair includes supporting the macrophage transition towards an anti-inflammatory macrophage phenotype [124], as IL-4-secreting eosinophils support skeletal and liver tissue repair/regeneration [200,207]. However, whether IL-4-secreting eosinophils facilitate tissue repair by supporting an anti-inflammatory leukocyte environment has yet to be tested. 

Several clinical and animal model studies implicate recruited DCs as critical regulatory factors for controlling the biphasic inflammatory response to cardiac damage. Previously, infarcted myocardial tissue from AMI patients revealed a significant elevation in DCs, macrophages, and T cells in the infarct region [127,131]. Moreover, immunostaining of patient infarcts demonstrated contacts between DCs and T cells, suggesting direct activation of cardiac T cells by DCs [131]. Similarly, recruited rat DCs associate with and activate T lymphocytes, particularly T helper cells, at the border region [133]. Activated DCs also migrate from the infarct region to the pericardial adipose tissue, where they proliferate and release various cytokines for T-cell expansion and granulopoiesis (production of neutrophils, basophils, and eosinophils) [153]. Specific depletion of cDCs (Zbtb26-DTR) after cardiac injury prevented harmful inflammatory responses, with lowered infiltration of neutrophils, macrophages, and T cells. This inhibition of inflammatory leukocyte recruitment improved cardiac function and limited adverse cardiac remodeling [136]. The destructive effects of cDC infiltration after injury may result from cDCs activating cytotoxic CD8+ T cells towards cardiac antigens, resulting in persistent damage to the myocardium [95,137]. DC loss also led to enhanced recruitment of classical pro-inflammatory monocytes and macrophages with impaired recruitment of anti-inflammatory/reparative monocytes and macrophages [139]. 

B-cell subtypes also have vital roles in regulating the inflammatory environment after cardiac injury. Activated B cells are responsible for secreting Ccl7, which induces pro-inflammatory Ly6C^hi^ monocytes to mobilize to the injury site for an elevated period of myocardial inflammation [152]. Further, cardiac injury enhances the number of granulocyte-macrophage colony-stimulating factor (GM-CSF)-producing B-cell clusters within the pericardial adipose tissue. These GM-CSF B cells promote the expansion of DCs and T cells in the pericardial adipose tissue as well as cardiac neutrophil infiltration after injury [153]. Knockout of IL-10-producing B cells led to aggravated cardiac injury and impaired cardiac function, due to delayed resolution of inflammation from retention of Ly6C^hi^ monocytes [156]. The beneficial effects of Breg transfer were also attributed to their secretion of IL-10, which limits CCR2 expression on monocytes and is required for the mobilization of Ly6C^hi^ monocytes from the bone marrow or blood to the heart [157]. As monocyte-derived macrophages (CCR2+) remain in the myocardium for weeks after injury and exacerbate adverse cardiac remodeling [75,77,78,79,80,81], this study implicates Bregs as a potential therapeutic candidate. 

T-cell subtypes also control the inflammatory response after cardiac injury through their interactions with other leukocytes. Heart failure patient biopsies revealed enhanced numbers of CD4+ Th1 and CD8+ T cells. These T cells were most likely activated as they co-localized with APCs [95,96]. Cardiac repair requires T-cell activation, as depletion of APCs such as DCs contributes to adverse cardiac remodeling [139]. CD4 knockout mice significantly recruit Ly6C^hi^ monocytes to the infarct and inhibit repair with altered collagen deposition and neovascularization [88]. Adult mice depleted of cytotoxic CD8+ T cells exhibited improved cardiac repair capabilities from macrophage polarization towards an anti-inflammatory reparative phenotype [103,104,105]. Treg cell loss significantly promotes pro-inflammatory leukocyte recruitment, with enhanced numbers of neutrophils, Ly6C^hi^ monocytes, CD4+ and CD8+ T cells, and inflammatory macrophages to the infarcted region [100,109,110,111]. In regenerative systems, Tregs prevent fibrosis through macrophage phenotype polarization away from a pro-fibrotic, inflammatory subtype in neonatal mice and zebrafish [116,118]. 

## 5. Conclusions

Current therapies after MI prevent further loss of ischemic tissue, but these methods are incapable of restoring the lost myocardium, leading to the development of a non-contractile scar and heart failure (Figure 1) [6,7,8]. Low rates of cardiomyocyte turnover throughout life and evidence for mammalian neonatal cardiac regeneration suggest the presence of endogenous regenerative mechanisms in mammals [9,10,11,12,13,14]. As these innate mechanisms are lost or inhibited with age, we can examine regenerative pathways in neonatal mice and adult zebrafish which display robust regeneration after cardiac injury (Figure 1) [20,21,22,23,24,40,41,42]. Further, differences in response to cardiac injury result from the differential recruitment strength and persistence of various innate and adaptive leukocytes in non-regenerative vs. regenerative systems (Figure 2, Table 1). In particular, significant levels of neutrophils, monocytes/macrophages, and T cells remain and contribute to adverse cardiac remodeling after heart injury in non-regenerative systems in comparison to resolution of these cells within 14 days in regenerative systems. We also see disparities in recruitment strengths between non-regenerative and regenerative models, with some cells experiencing diminished recruitment in regenerative systems (neutrophils, T cells) or stronger mobilization (monocytes/macrophages) in comparison to non-regenerative adult or juvenile mice. However, there are still significant knowledge gaps on leukocyte function after myocardial infarction in both non-regenerative and regenerative systems. Therefore, with this review, we aim to inspire future research in the cardiac injury and regenerative field. 

Additional research is needed to further characterize and identify critical immune cells and their subtypes in each model system. Conflicting analyses on the leukocyte response in humans after myocardial infarction (Table 1 and Table 2) result from a lack of standardized markers for phenotyping and the timing of examination of patient peripheral blood. Further, clinical studies only reveal a small fragment of the leukocyte story during MI. Analyses of collected blood samples represent a small subset of the total immune cell population and fail to indicate conclusively whether the leukocyte in question has mobilized to the infarcted myocardium. Accordingly, most of our understanding of the immune response after cardiac injury derives from investigations in animal model systems. However, previous animal studies focused on only one leukocyte in isolation and whether loss of this cell type affected cardiac scarring, function, or regeneration after injury. These reports also failed to examine the complete leukocyte response by examining the effect of depletion of one immune cell type on the recruitment and persistence of other leukocytes. Additionally, as each leukocyte is composed of heterogeneous cell populations, with potentially different time courses and functionalities in response to cardiac injury, the current information available on leukocyte roles after MI is significantly lacking. Further, results from previous studies may also need to be re-examined and similar studies conducted again using more in-depth analyses, as examining one cell type in isolation provides misleading and incomplete information, leading to potential misinterpretation of the data and inconsistent observations between different studies. Therefore, there is little to no information available on immune cell interactions during cardiac repair or regeneration in non-regenerative and regenerative systems, respectively. 

At present, there are no available markers or antibodies to examine several leukocyte types in the zebrafish model: dendritic cells, basophils, and natural killer-like cells. Moreover, current methods to analyze macrophages in zebrafish are non-specific, as the well-utilized *mpeg1.1* reporter also labels B cells and natural killer-like cells [182,183]. Further, leukocytes form incredibly complex and diverse heterogenous cell populations, as observed with the nearly 300 different macrophage transcriptomes revealed in response to various stimuli [179]. Currently, only the Treg subtype has genetic tools available to study this cell type in isolation (*foxp3a*) in zebrafish [118]. Therefore, markers to examine other T-cell, macrophage, and neutrophil subtypes in isolation are also needed to further elucidate their roles in cardiac repair and regeneration in the zebrafish model. 

To overcome these limitations and develop better genetic tools and prognostic markers, single-cell transcriptome analyses on leukocytes isolated from steady-state and cardiac injury hearts should be conducted. Further, comparisons of the heart immune cell transcriptome between non-regenerative adult humans and mice and regenerative neonatal mice and adult zebrafish will expand our current understanding of the role of each leukocyte in tissue repair and regeneration. Overall, in-depth analyses of these transcriptome datasets will enable the identification of specific leukocyte subtype markers for the development of novel genetic tools and uncover essential immune cell type similarities and differences. Discrepancies in the genetic profiles of leukocytes purified from non-regenerative vs. regenerative animal model systems may be exploited to develop new therapies capable of stimulating endogenous regenerative mechanisms after ischemic injury in humans.

## Figures and Tables

**Table 1:** Leukocyte functions after heart damage in non-regenerative vs. regenerative models.

Leukocyte	Humapeak 3 Days with Permanent Ligation; Days 1ns	Adult Rodents	Neonatal Mice	Adult Zebrafish
**Neutrophils**	**Detrimental**: Neutrophilia associated with increased infarct size, heart failure, death [49,50,51,52,53,54,55,56].	**Timing:** Days 1–14, peak 3 days with permanent ligation; days 1–7, peak day 1 with reperfusion [57,58]. **Detrimental:** CM death and adverse cardiac remodeling [59,60,61]. **Beneficial:** Clear dead cells/debris, angiogenesis, inflammation resolution [60]; reparative macrophage polarization [62].	**Timing:** Overall larger neutrophil recruitment in P1 neonates vs. P14 juveniles [63,64].	**Timing:** From 6 h to 7 days; peaks at day 1, drops days 3–7, basal levels day 14 after cryoinjury [65,66,67,68]. **Detrimental:** Delayed removal extends inflammatory phase and inhibits regeneration with scar retention, lowered CM proliferation [65,67]. **Beneficial:** Support angiogenesis [67].
**Monocytes/** **Macrophages**	**Timing:** At infarct border region (12 h-5 days post-MI); and infarct (5–14 days post-MI) [69]. **Detrimental:** Pro-inflammatory monocytes and adverse recovery [70,71,72,73,74]; pro-inflammatory monocyte-derived CCR2+ macrophages accumulate in heart failure patients with LV remodeling [75].	**Timing:** Biphasic with days 1–3 pro-inflammatory Ly6C^hi^ cells; day 5 onward reparative Ly6C^lo^ cells; remain at 14 days after injury [57,76]. **Detrimental:** CCR2+ macrophages persist in myocardium for months, role in ventricular remodeling [77,78,79,80,81]. **Beneficial:** Clear cell debris/dead cells and neutrophils; angiogenesis; and collagen deposition [82]; early phase for cell clearance; late phase for tissue granulation/collagen deposition [76]; tissue-resident CCR2- cardioprotective [77,78,79,80,81].	**Timing:** Biphasic with pro-inflammatory and then anti-inflammatory cells; regenerative P1 higher levels than non-regenerative P14 juveniles [64]. **Beneficial**: Depletion abolishes regeneration with scar retention, lowered angiogenesis, and cardiac remodeling [63,64]; neonates expand tissue-resident CCR2- population and do not recruit CCR2+ monocytes after injury [63].	**Timing:** Biphasic recruitment of pro-inflammatory TNFα+ (1–3 dpi) and anti-inflammatory TNFα- (3–7 dpi) after injury; returns to basal levels by 14 dpi [68,83]. **Beneficial:** Depletion or delayed mobilization inhibits neutrophil clearance, CM proliferation, scar resolution, and angiogenesis [65,66,67,68,84]; reparative *wt1b*+ macrophages support CM proliferation [85].
**T lymphocytes**	**Timing:** Decrease in CD4+ and CD8+ T cells immediately after AMI with increase in Tregs 4–6 days later [86,87,88]; increase in Th1 and Th17 CD4+ T cells with decrease in Tregs [89,90,91,92]. **Detrimental:** CD8+ CD28+ T cells lowered cardiac function [93]; CD8 + CD57+ T cells correlate with mortality [94]; heart failure patients enriched with CD4+ Th1 and CD8+ T cells [95,96]. **Beneficial:** Low Treg levels associated with LV remodeling, and lowered survival [97,98].	**Timing:** T cells mobilize to injured heart from days 1 to 14, peaking at day 7 post-injury with permanent ligation, day 3 with reperfusion [57,88,99,100,101,102]; remain for weeks after injury [102]. **Detrimental:** CD4+ cells aggravate injury [99]; CD8+ cells enhance inflammation and CM apoptosis [103,104,105]; γδ cells promote inflammation, CM death, fibrosis, adverse remodeling [106,107]. **Beneficial:** CD4+ for collagen deposition, neovascularization, cardioprotective phenotype [88,108]; Tregs in wound recovery, resolve inflammation [100,109,110,111,112,113,114,115]; CD8+ to prevent cardiac rupture [104,105].	**Timing:** T cells mobilize from days 1 to 14, peak at day 7 post-injury, return to basal levels day 14 in P3 mice; significantly higher CD4+ T cells in P8 juveniles vs. P3 neonates, with T-cell persistance high through day 14 [116]. **Detrimental:** Depletion of CD4+ T cells in P8 juveniles facilitated regeneration with CM proliferation and reduced fibrosis [117]. **Beneficial:** Tregs support regeneration in neonates with CM proliferation and reduced cardiac fibrosis [116].	**Timing:** T cells mobilize to cardiac wound from day 1, peak at 7 dpi, resolved by 14 dpi [68,118]. **Beneficial:** Depletion of Tregs during cardiac cryoinjury led to thinner myocardial walls, persistent collagenous scar, lowered CM proliferation, and macrophage polarization towards classical inflammatory phenotype [118].

Abbreviations: MI, myocardial infarction; MACE, major adverse cardiovascular events; AMI, acute myocardial infarction; CM, cardiomyocyte; LV, left ventricular; P1, 1 day postpartum; P3, 3 days postpartum; P8, 8 days postpartum; P14, 14 days postpartum; dpi, days post-injury; TNFα, tumor necrosis factor α; IL-2, interleukin-2; IL-10, interleukin-10; IL-17A, interleukin-17A.

**Table 2:** Leukocyte functions after heart damage only examined in non-regenerative systems.

Leukocyte	Humans	Adult Rodents
**Eosinophils**	**Detrimental:** Lower peripheral blood levels associated with increased risk for MACE and death [119,120,121,122,123,124]. **Beneficial:** Enhanced levels cardioprotective: limit CM death, fibrosis, and inflammation [125]; protection 6 months post-reperfusion [123].	**Timing:** Mobilize from days 1 to 7, peak at day 4 post-injury, lower at day 7 [125]. **Beneficial:** Depletion causes severe cardiac dysfunction after MI with larger infarct, increased cell death and collagen deposition [124,125].
**Basophils**	**Timing:** Peak at 96 h post-MI [126]. **Detrimental:** Both high and low counts associated with higher mortality [122]. **Beneficial:** Lower numbers 1 week post-MI associated with larger infarct and adverse cardiac outcomes [126].	**Timing:** Mobilize from days 1 to 7, peak between days 3 and 7 post-injury, return to baseline by day 14; infiltrated infarcted hearts [126]. **Beneficial:** Depletion leads to severe cardiac dysfunction; enhanced pro-inflammatory Ly6C^hi^ monocytes and lowered anti-inflammatory macrophages [126].
**Dendritic Cells**	**Timing/Localization:** Decrease in patients with AMI; increase in activated DCs [127,128,129,130]; significant elevation in DCs in infarcted myocardium; interaction of infiltrated DCs with T cells [127,131]. **Beneficial:** Cardioprotective, lower amounts of DCs in infarcted myocardium associated with enhanced macrophages, limited fibrosis, and cardiac rupture [132].	**Timing:** Accumulate from day 1 post-injury, peak at day 7, and lowered at day 14 with permanent ligation; peaked day 3 with reperfusion and lowered by day 7 [57,133]. **Detrimental:** Mature DCs worsen ventricular remodeling [134]; prevention of DC mobilization from bone marrow improved cardiac function [135]; depletion of cDCs limited inflammatory response with lowered neutrophil, macrophage, and T-cell infiltration and reduced adverse remodeling [136]; cross-priming cytotoxic T cells for perpetuation of myocardial damage [95,137]; pDCs release IFN-γ and further damaging inflammatory responses [138]. **Beneficial:** DC depletion led to reduced cardiac function and adverse remodeling; required for recruitment of anti-inflammatory reparative macrophages [139]; DC depletion led to larger infarcts, severe cardiac remodeling, and dysfunction [140].
**Natural Killer** **Cells**	**Timing/Localization:** Either elevated or lowered levels in peripheral blood [141,142,143]; infiltrate myocardium [142]; defective functionalities, reduction in cytotoxicity after AMI [143,144,145]. **Beneficial**: IL-10+ NK cells 72 h post-MI; reduced IL-10+ NK cells associated with enhanced functional recovery 3 months post-MI [143].	**Timing:** Mobilize from days 1 to 7, peak day 7 with permanent ligation, and lowered at day 14; peaked day 3 with reperfusion, lowered day 7 [58,59,146]. **Beneficial:** Mobilization to infarct improves cardiac function and remodeling [146]; IL-2-activated NK cells support angiogenesis and reduce fibrosis [147,148]. **Detrimental:** NK depletion before injury led to reduced infarct and limited adverse remodeling [149].
**B lymphocytes**	**Timing/Localization:** B cells present in non-ischemic hearts [150]; decrease after AMI and increase after reperfusion [86]; intravascular B cells that associate with cardiac endothelium [151].	**Timing:** Mobilize from days 1 to 7, peak at day 7 post-injury, and lowered at day 14 with permanent ligation; peaked at day 3 and lowered day 7 with reperfusion [57]. **Detrimental:** B2 cell depletion limits cardiac injury, prevents adverse remodeling, and enhances cardiac function; activated B2 cells secrete Ccl7 for pro-inflammatory Ly6C^hi^ monocytes and to extend inflammation [152]; enhanced B cells associated with increased cardiac fibrosis and remodeling; GM-CSF-producing B cells promote DC and T-cell expansion in PAT and neutrophil infiltration to infarct [153]; B-cell knockout system lowered pro-inflammatory cytokine levels, ventricular remodeling, fibrosis [154]. **Beneficial:** B-cell injections improved cardiac function, reduced apoptosis [155]; IL-10-producing B1a cells required for inflammation resolution [156]; Breg adoptive transfer enhances cardiac function through IL-10 secretion to limit mobilization of CCR2+ Ly6C^hi^ monocytes to heart [157].

Abbreviations: MI, myocardial infarction; MACE, major adverse cardiovascular events; AMI, acute myocardial infarction; CM, cardiomyocyte; LV, left ventricular; DC, dendritic cell; NK, natural killer; dpi, days post-injury; IL-2, interleukin-2; IL-10, interleukin-10; IL-17A, interleukin-17A; GM-CSF, granulocyte-macrophage colony-stimulating factor; PAT, pericardial adipose tissue.

## Data Availability

Not applicable.
